# Disentangling the effects of photosynthetically active radiation and red to far-red ratio on plant photosynthesis under canopy shading: a simulation study using a functional–structural plant model

**DOI:** 10.1093/aob/mcz197

**Published:** 2019-12-03

**Authors:** Ningyi Zhang, Arian van Westreenen, Niels P R Anten, Jochem B Evers, Leo F M Marcelis

**Affiliations:** 1 Horticulture and Product Physiology Group, Department of Plant Sciences, Wageningen University, 6700 AA Wageningen, The Netherlands; 2 Centre for Crop Systems Analysis, Department of Plant Sciences, Wageningen University, 6700 AK Wageningen, The Netherlands

**Keywords:** Canopy architecture, functional–structural plant model, light interception, phenotypic plasticity, photosynthesis, red to far-red ratio, rose (*Rosa hybrida*), shade

## Abstract

**Background and Aims:**

Shading by an overhead canopy (i.e. canopy shading) entails simultaneous changes in both photosynthetically active radiation (PAR) and red to far-red ratio (R:FR). As plant responses to PAR (e.g. changes in leaf photosynthesis) are different from responses to R:FR (e.g. changes in plant architecture), and these responses occur at both organ and plant levels, understanding plant photosynthesis responses to canopy shading needs separate analysis of responses to reductions in PAR and R:FR at different levels.

**Methods:**

In a glasshouse experiment we subjected plants of woody perennial rose (*Rosa hybrida*) to different light treatments, and so separately quantified the effects of reductions in PAR and R:FR on leaf photosynthetic traits and plant architectural traits. Using a functional–structural plant model, we separately quantified the effects of responses in these traits on plant photosynthesis, and evaluated the relative importance of changes of individual traits for plant photosynthesis under mild and heavy shading caused by virtual overhead canopies.

**Key Results:**

Model simulations showed that the individual trait responses to canopy shading could have positive and negative effects on plant photosynthesis. Under mild canopy shading, trait responses to reduced R:FR on photosynthesis were generally negative and with a larger magnitude than effects of responses to reduced PAR. Conversely, under heavy canopy shading, the positive effects of trait responses to reduced PAR became dominant. The combined effects of low-R:FR responses and low-PAR responses on plant photosynthesis were not equal to the sum of the separate effects, indicating interactions between individual trait responses.

**Conclusions:**

Our simulation results indicate that under canopy shading, the relative importance of plant responses to PAR and R:FR for plant photosynthesis changes with shade levels. This suggests that the adaptive significance of plant plasticity responses to one shading factor depends on plant responses to the other.

## INTRODUCTION

Phenotypic plasticity in plants is their ability to change their phenotype according to the environmental conditions in which they grow (Bradshaw, 1965; Schlichting, 1986; Sultan, 2000). Analysing plant plastic responses to environmental conditions and their subsequent effects on plant performance is complicated, particularly because (1) phenotypic plasticity often includes changes in multiple interacting functional traits, and (2) changes in environmental conditions typically include changes in multiple factors that can induce different phenotypic responses and thus influence plant performance in different ways (Callaway *et al*., 2003; Anten *et al*., 2010).

An example of analysing the complex consequences of plant plasticity for plant performance is analysing the effects of phenotypic plasticity to shading caused by leaves (i.e. canopy shading) on plant photosynthesis, which is an important plant performance measure. Plants grow in dynamic vegetation stands with other growing plants where they shade one another creating a light environment that varies considerably in time and space. This canopy shading entails multiple factors including reductions in photosynthetically active radiation (PAR) and changes in spectral composition especially reductions in the red (655–665 nm) to far-red (725–735 nm) ratio (R:FR) in addition to other spectral changes (Smith, 1982). Reductions in PAR and R:FR occur simultaneously but at different magnitudes with increasing level of canopy shading. Reductions in PAR and R:FR also induce different plant plastic responses. Reductions in PAR, on the one hand, directly decrease plant photosynthesis due to reductions in light as a resource. On the other hand, reductions in PAR induce plastic responses such as decreasing leaf photosynthetic capacity and respiration rate, changing leaf anatomy, increasing leaf photosynthetic nitrogen, increasing specific leaf area and increasing the fraction of assimilates partitioned to the leaf (Gulmon and Chu, 1981; Lichtenthaler *et al*., 1981; Walters *et al*., 1993; Evans and Poorter, 2001; Baird *et al*., 2017). Reductions in R:FR mostly induce plastic responses of plant morphogenesis, including increased elongation of hypocotyls, internodes, petioles, leaf sheaths and blades, increased leaf inclination angle, reduced branching and tillering, and early flowering (Franklin, 2008; Casal, 2012; Pierik and De Wit, 2014). These plastic responses to reductions in PAR and R:FR may affect plant photosynthesis in different directions (i.e. positive and negative) and with different magnitudes. For instance, the decrease of respiration rate caused by low PAR may have a positive effect on plant photosynthesis, whereas the increase of leaf angle caused by low R:FR may decrease plant photosynthesis due to reduced light interception. Moreover, the effects of individual plastic responses on plant photosynthesis may interact with each other. For instance, plant photosynthesis can be affected by responses in both plant architecture (affecting plant light capture) and leaf photosynthetic traits (affecting plant light use); these responses interact as effects of changes in leaf photosynthetic traits depend on the amount of light that the leaf receives.

To fully understand the effect of phenotypic plasticity on plant photosynthesis under canopy shading, we need to (1) determine individual trait responses to each factor (reduced PAR and reduced R:FR) separately, (2) determine how these trait effects change with shading levels and (3) quantify how the syndrome of trait responses to these shading factors in coherence determine plant photosynthesis. Several studies have quantified the effects of some plastic responses of individual traits (e.g. longer petiole length and larger specific leaf area) on plant performance under low PAR or low R:FR (Weijschedé *et al*., 2006; Liu *et al*., 2016). However, as far as we know, no study has separately quantified the effects of multiple plastic trait responses induced by low PAR and low R:FR and compared their relative importance for plant photosynthesis under canopy shading. These analyses cannot be done by experiments alone, because it is impossible to induce each plastic trait response independently while preventing the expressions of other traits in real plants. Creating virtual plants by models can be very helpful in addition to experiments, because models allow us to combine any trait in virtual phenotypes. Functional–structural plant (FSP) models simulate plant growth in response to environmental changes taking into account both plant architecture in three dimensions and physiological processes such as photosynthesis, respiration and biomass allocation (Vos *et al*., 2010). Thus, FSP models can be used to disentangle individual trait responses to canopy shading and to separately quantify the effects of individual trait responses on plant photosynthesis. By creating virtual phenotypes in which individual plastic traits are varied one by one, the effect of each plastic trait on plant photosynthesis can be assessed separately (Bongers *et al*., 2014). Subsequently, the interactive effects of several trait responses on plant photosynthesis can be quantified.

The objective of this study was to quantify the extent to which plastic responses to different shading factors interact in determining plant photosynthesis under different levels of canopy shading. First, the separate plastic responses to two main factors of canopy shading (reduced PAR and reduced R:FR) were assessed experimentally. Then, an FSP model was applied to quantify the effects of individual plastic responses and the consequences of their interactions for plant photosynthesis under different shading levels. To this end, a glasshouse experiment was conducted in which plants of the woody perennial rose (*Rosa hybrida*) were subjected to different light treatments: reductions in only PAR and R:FR, and combinations of the two, to evaluate plant plastic responses of plant architecture and leaf photosynthesis to reduced PAR and reduced R:FR. We chose rose as an example to study shade responses because, in rose production, both lighting and plant architecture are often manipulated intensively (Gonzalez-Real and Baille, 2000). An FSP model of rose was then developed and validated using the experimental data, and the model was used to conduct simulation studies to quantify the effect of each trait response on light interception and plant photosynthesis.

## MATERIALS AND METHODS

### Plant materials and growth conditions

The experiment was conducted in two neighbouring compartments (8 × 8 m) of a Venlo-type glasshouse located in Wageningen, the Netherlands (52°N, 6°E). The environmental conditions in the two compartments were similar. In each compartment, there were four rolling growth tables (1.70 × 3.25 m). Rose plants (*Rosa hybrida* ‘Red Naomi!’) with one-node cuttings bearing a shoot were grown in rockwool cubes (0.1 × 0.1 × 0.1 m). On 5 April, 2016, plants were placed on growth tables with a row distribution. The distance between each row was 0.15 m. The distance between each plant within a row was also 0.15 m. When on average one flower bud had just appeared per plant, the shoots were pruned just above the third five-leaflet leaf and this leaf was removed to stimulate axillary bud break, as rose growers commonly do in practice. We started the light treatments 1 week after pruning, when the axillary buds were broken (average shoot length of 1 cm). The experiment lasted for 6 weeks and finished by the end of May 2016.

The experiment had a one-factorial design with six light treatments (Table 1). The treatments were established as a randomized block design with four blocks, with 100 plants in each treatment plot (1.7 × 1.6 m). The randomized block design was used to take into account possible gradients in the glasshouse environment and the differences in time of measurements. In each plot, two rows of plants on each side of the plot were used as border plants that were not included in measurements. Light treatments included reductions in PAR and reductions in R:FR, and a combination of the two (Table 1). Because the light treatments aimed at mimicking the situation whereby our target plants are shaded by an overhead canopy, which reduces the level of PAR and R:FR above the target plants, light conditions above the plant canopy were manipulated in the treatments.

**Table 1.** The relative levels of photosynthetically active radiation (PAR) and red to far-red ratio (R:FR) achieved in different light treatments

Treatments		Relative levels (%)	
PAR	R:FR	PAR	R:FR
High	High*	100	100
High	Medium	100	34
High	Low*	100	24
Medium	High*	60	90
Medium	Low*	70	15
Low	High	29	89

Percentage values were calculated as the PAR and R:FR measured in each treatment divided by the PAR and R:FR measured in the high PAR and high R:FR treatment. Plant architectural measurements were conducted in all six treatments. Leaf photosynthetic measurements were conducted in treatments denoted with an asterisk.

Reductions in PAR were achieved by adding neutral shading net (50 % transmissivity) at a distance of 20 cm above the canopy, without changing the R:FR level. One layer of neutral shading net was added in the ‘medium PAR + high R:FR’ treatment and two layers of neutral shading net were added in the ‘low PAR + high R:FR’ treatment. These two treatments were used to quantify plant responses to reductions in PAR only. Reductions in R:FR were achieved by adding additional far-red light-emitting diode (LED) modules (GreenPower far-red production modules, Philips, Eindhoven, the Netherlands) at a distance of 50 cm above the canopy, without changing the PAR level. This allowed us to manipulate R:FR independent of PAR. Two LED modules per plot were added in the ‘high PAR + medium R:FR’ treatment, resulting in an additional 50 μmol m^−2^ s^−1^ far-red light intensity from LED modules at the canopy level. Four LED modules per plot were added in the ‘high PAR + low R:FR’ treatment, resulting in an additional 100 μmol m^−2^ s^−1^ far-red light intensity from LED modules at the canopy level. These two treatments were used to quantify plant responses to reductions in R:FR only. In addition, there was a treatment without reductions in PAR and R:FR (i.e. ‘high PAR + high R:FR’ treatment), to represent the non-shaded condition, and a treatment with reductions in both PAR and R:FR (i.e. ‘medium PAR + low R:FR’ treatment), to mimic the situation of shading by an overhead canopy, which causes reductions in both PAR and R:FR. A combination of low R:FR and medium PAR was used because even a moderate canopy shading causes a relatively strong reduction of R:FR even when the reduction of PAR is still relatively minor (Ballaré, 1999). This treatment was achieved by adding one layer of neutral shading net and four LED modules above the canopy. In treatments where there were fewer than four LED modules in a plot, we added fake modules to ensure similar shading by the frame of LED modules for all treatments. Each treatment plot was surrounded by plastic film (with white colour facing the plot and black colour facing outside) with 20 cm depth from the top of LED frames to minimize light treatments affecting each other. The actual levels of PAR and R:FR achieved in different treatments are given in Table 1. PAR levels were measured using a line quantum sensor (LI-191R, LiCor, Lincoln, NE, USA). R:FR ratios were measured using a spectrometer (SpectroSense2 system, Skye Instruments Ltd, UK) which measures the red and far-red light coming from different directions within 180°. In addition, light spectrum (400–800 nm) measurements were conducted for the ‘high PAR + high R:FR’, ‘high PAR + low R:FR’, ‘medium PAR + high R:FR’ and ‘medium PAR + low R:FR’ treatments using a spectrometer (Ocean Optics, Inc., Largo, FL, USA) (Supplementary Data Fig. S1).

Assimilation lighting (high-pressure sodium lamps, Philips, Eindhoven, the Netherlands) was only used to prevent assimilation lighting of neighbouring compartments disturbing the treatments and to prevent gradients within the compartments. During the experimental period, the assimilation lighting was on for approximately 6 h per day with an intensity of 150 µmol m^−2^ s^−1^. The daily average light intensity (including both sunlight and assimilation lighting) during the light period inside the glasshouse in the experiment is given in Supplementary Data Fig. S2. In addition, R:FR ratios under different numbers of additional far-red lamps with the assimilation lighting on or off are given in Table S1. The set point of relative humidity was 65 % during day and night. Set points of day and night temperature were 22 and 17 °C, respectively. The daily average relative humidity and day and night temperature inside the glasshouse during the experimental period are given in the Supplementary Data (Fig. S2). CO_2_ was not controlled. Watering (EC = 1.6 mS cm^−1^; pH 6) was done with an ebb and flood system four times a day.

### Measurements

#### Plant architecture measurements.

 In each plot, five plants were randomly chosen to measure plant architectural traits when flower buds started to open. The measurements were conducted on 19, 20, 21 and 23 May, 2016, in which measurements for all plants in one block were finished per day. First, measurements of plant height, leaflet number of every leaf and leaf inclination angle of every second leaf were made non-destructively. Plants were then dissected to measure internode length and leaf area of every second internode or leaf, plant total leaf area, peduncle length and flower bud diameter. Length measurements were made using a ruler. Leaf inclination angle was measured as the insertion angle of the leaf compared with the horizontal level using a protractor. Leaf area was measured using a leaf area meter (LICOR-3100, LiCor). Flower bud diameter was measured using a caliper.

#### Leaf optical property measurements.

Leaf reflectance and transmittance were measured in the range 400–700 nm for both adaxial and abaxial sides of a leaf. The measurement system consisted of two integrating spheres, each connected to a spectrometer and an LED light source. Details of the measurement system were described in Hogewoning *et al*. (2010). In each treatment, two plants (with one plant from each experimental compartment) were taken for the measurements. In each plant, the top leaf was used, and three leaf discs (with a diameter of 1 cm) were taken from the terminal leaflet of the top leaf.

#### Leaf gas exchange measurements.

As leaf gas exchange measurements are time-consuming, four out of six treatments were chosen to conduct this type of measurement. The four treatments were high PAR + high R:FR (representing the case of no canopy shading), medium PAR + high R:FR (representing the case where only PAR was reduced by canopy shading), high PAR + low R:FR (representing the case where only R:FR was reduced by canopy shading) and medium PAR + low R:FR (representing the case where PAR and R:FR were simultaneously reduced by canopy shading). Two plants in each plot were randomly chosen at the flower bud visible stage to perform a combined measurement of gas exchange and chlorophyll fluorescence using the LI-6400XT Portable Photosynthesis System (Li-Cor) on leaves at the upper, middle and lower level of the canopy. The measurement was conducted on the terminal leaflet of each leaf. Light response curves of photosynthesis were made by decreasing incident light in the leaf cuvette in the series 1500, 1200, 1000, 750, 550, 350, 200, 150, 100, 50, 20 and 0 µmol m^−2^ s^−1^, while keeping ambient CO_2_ at 400 µmol mol^−1^, leaf temperature at 25 °C, and leaf-to-air vapour pressure difference at 1–1.6 kPa. Steady-state fluorescence (*F*_s_) was measured simultaneously with the gas exchange measurement after 3–5 min of light adaptation, followed by applying a light pulse >8000 µmol m^−2^ s^−1^ for less than 1 s to measure maximum fluorescence $F_{m}^{\prime }$

### Estimation of photosynthetic parameters

Leaf photosynthetic parameters were estimated by stepwise fitting the combined measurements to a non-rectangular hyperbola (eqn 1) (Marshall and Biscoe, 1980):

$$\begin{array}{l} A=\frac{\Phi_{CO2LL(inc)}I_{inc}+A_{max}-\sqrt{{\left(\Phi_{CO2LL(inc)}I_{inc}+A_{max}\right)}^{2}-4\theta A_{max}\Phi_{CO2LL(inc)}I_{inc}}}{2\theta }-R_{d} \end{array}$$ (1)

where *A* (μmol CO_2_ m^−2^ s^−1^) is the net leaf photosynthetic rate; *Φ*_CO2LL(inc)_ (mol CO_2_ mol^−1^ photon) is the quantum yield of CO_2_ assimilation on the basis of incident light; *I*_inc_ (μmol m^−2^ s^−1^) is the incident irradiance; *A*_max_ (μmol CO_2_ m^−2^ s^−1^) is the maximum leaf photosynthetic rate at saturating incident irradiance; *θ* is the curvature factor of the light response curve; and *R*_d_ (μmol CO_2_ m^−2^ s^−1^) is the dark respiration rate. Details on the procedure for estimating *Φ*_CO2LL(inc)_, *A*_max_, *θ* and *R*_d_ can be found in Supplementary Data Method S1.

*R*_d_ was assumed to be linearly related to *A*_max_ and this linear relationship was quantified by curve fitting eqn (2) (Hikosaka *et al*., 2016) using the estimates of *A*_max_ and *R*_d_ (in eqn 1) for top, middle and low leaves in the canopy:

$$\begin{array}{l} R_{d}=s\times A_{max} \end{array}$$

where *s* is the slope of the linear relationship between *A*_max_ and *R*_d_.

To calculate photosynthesis at the plant level, photosynthetic parameters of individual leaves of the plant need to be estimated. According to Niinemets and Anten (2009), *A*_max_ of a leaf in the canopy can be corelated with the relative light level experienced by that leaf (eqn 3):

$$\begin{array}{l} A_{max,0}=A_{max,top}\times {\left(Q_{0}/Q_{top}\right)}^{k} \end{array}$$

where *A*_max,0_ (μmol CO_2_ m^−2^ s^−1^) is the *A*_max_ of an individual leaf in the canopy; *Q*_0_ (μmol m^−2^ s^−1^) is the light intensity at the level of that specific leaf; *Q*_top_ (μmol m^−2^ s^−1^) is the light intensity on top of the canopy; *Q*_0_/*Q*_top_ gives the relative light intensity experienced by that specific leaf; *A*_max,top_ (μmol CO_2_ m^−2^ s^−1^) is the *A*_max_ of the top leaf in the canopy; and *k* is the coefficient describing the relationship between light gradient and *A*_max_ gradient in the canopy. To estimate the value of *k* in eqn (3), light intensities at the top, middle and lower levels of the canopy were measured on a cloudy day (in which incoming light was evenly distributed) at flowering stage using a line quantum sensor (Li-Cor). Relative light intensities at these levels were then calculated. Based on the estimated *A*_max_ values of top, middle and low leaves and the calculated relative light intensities experienced by these leaves (Supplementary Data Fig. S3), *k* was estimated by curve fitting eqn (3).

### Statistical analysis

The six treatments were considered as independent fixed factors. Treatment effects on plant architectural traits and leaf photosynthetic parameters were analysed using a one-way ANOVA (*P* < 0.05) of R (version R 3.5.0, R Foundation for Statistical Computing, Vienna, Austria), taking into account the block effects. Treatment effects on the slope *s* and coefficient *k* were tested by comparing whether the same regression model could be used in different treatments using an *F*-test (*P* < 0.05) of SAS (SAS Institute Inc., Cary, NC, USA).

### Simulations

#### Model development.

An FSP model of rose was developed to quantify the effect of trait responses to low PAR and low R:FR on light interception and plant photosynthesis under canopy shading. The model was constructed in the plant modelling software GroIMP (Hemmerling *et al*., 2008). The model includes (1) a static representation of the three-dimensional (3D) architecture of rose plants in a canopy at flowering stage, (2) a radiation model to simulate light capture of individual organs and (3) a photosynthesis model to calculate net daily photosynthesis of the whole plant.

#### (1) The 3D rose plants.

Plant 3D architecture was represented using a repetition of basic units (i.e. phytomers), which consist of an internode and a compound leaf. Each phytomer was sequentially placed along the growth axis of the plant. The size of the phytomer was determined by actual plant architectural measurements, with the assumption that the phytomer size between every second phytomer can be linearly interpolated. The assumption of linear interpolation of organ size was validated using data measured on the same cultivar in another experiment (the experiment is described in Zhang *et al*., 2019; validation results are given in Supplementary Data Fig. S4). The orientation of the first leaf was randomized for the plants. Phyllotaxis was obtained from measurements on the same cultivar in another experiment (=192°) and was fixed for all simulated plants (the experiment is described in Zhang *et al*., 2019). Leaf insertion angle was assumed to follow a normal distribution according to the average leaf angle and standard deviation of all replicates in each treatments (Zhang *et al*., 2019). Leaflet number of each compound leaf on the plant was obtained from the actual plant architectural measurements (Fig. S5). For plants with a flower bud, a red sphere with the measured flower diameter was added on top of the 3D plant representation to mimic the similar shading from the flower.

#### (2) The radiation model.

The light environment was modelled using both a diffuse light dome with moderate gradation towards zenith and azimuthal uniformity and a direct light source spread over the solar path (Evers *et al*., 2010). Thus, the simulated light environment was similar to the conditions of incoming light in the glasshouse. To eliminate the border effects in the light environment, each plot of a simulated plant population was replicated ten times in the *x* and *y* directions for the light model calculations, resulting in averaged light conditions that were experienced by 100 copies of each individual plant population (de Vries *et al*., 2018). The amount of light reaching the 3D objects (e.g. internode and leaf) was simulated using a Monte-Carlo ray tracer embedded in GroIMP (Hemmerling *et al*., 2008). Light was simulated for individual wavebands of PAR, red and far-red. The light absorption of an individual organ was calculated based on the amount of PAR reaching that organ and the optical properties of that organ (Evers *et al*., 2010; Buck-Sorlin *et al*., 2011). Leaf PAR absorption was then used to calculate leaf photosynthesis. Leaf PAR reflectance and transmittance values are given in Supplementary Data Table S2. Because leaf optical parameters measured in different treatments were quite similar (Table S2), the same optical parameter values were used in all simulations. Stems were assumed to have the same reflectance as leaves but with no transmission.

#### (3) The photosynthesis model.

Plant net photosynthesis was calculated as the sum of net photosynthesis of each individual leaf. Leaf net photosynthesis was calculated using eqn (1) based on the photosynthetic parameters and light absorption of that leaf. Each leaf had its own photosynthetic parameters. The gradients of *A*_max_ and *R*_d_ for individual leaves in the canopy were simulated in the model. *A*_max_ and *R*_d_ of each individual leaf were calculated using eqns (2) and (3) based on the input parameter values of *s*, *k* and *A*_max,top_, and the relative light intensity reaching that leaf. However, we did not simulate the gradients of *Φ*_CO2LL(inc)_ and *θ* for individual leaves in the canopy, as we did not find any substantial differences of these two parameters between leaves at different canopy levels. Therefore, *Φ*_CO2LL(inc)_ and *θ* were kept at the same values for leaves on the same plant.

#### Model evaluation. 

The simulated fraction of light intercepted by the plants, plant height and plant total leaf area were compared with the measurements by calculating the coefficient of determination (*r*^2^) and the relative root-mean-square deviation (RMSD). Because our model was a static one in which plant 3D architecture was created based on organ-level measurements instead of converting from plant growth, we validated it using data obtained at the plant level (plant height and total leaf area) and canopy level (the fraction of light intercepted by the plants). The assumptions used in creating the 3D plant architecture, including the linear interpolation of organ size and the normal distribution of leaf inclination angle, were also tested by such a type of model validation.

#### Model simulation design. 

Plants were simulated at the stage when all leaves had reached their final area. A distance of 0.15 m between rows and individual plants in a row was used in the simulation (the same distance as in the experiment). A plant population on a 1-m^2^ soil area was simulated. The background incoming light intensity was set at 500 µmol m^−2^ s^−1^ and R:FR of the incoming light was set at 1.05, which respectively represented the measured average incident light level during the experiment and the incoming R:FR in the glasshouse.

In the simulations we created situations in which our target plants experience different levels of shading by an overhead canopy. However, because the focus of the model simulations was the target plants below the overhead canopy, the 3D shape of this overhead canopy was not simulated in the model. Light conditions of specific light treatments in the experiment (e.g. LEDs and the shading net used in the experiment) were not simulated either, as the canopy shading in the simulations did not necessarily correspond with the treatments in the experiment. Instead, the reductions of incoming PAR and R:FR by the overhead canopy were simulated based on empirical equations. Based on the relative reductions of PAR and R:FR by the overhead canopy, the corresponding plant phenotypes were created.

In total four levels of canopy shading were simulated, i.e. the leaf area index of the overhead canopy (LAI_C) was set at 0.5, 1, 2 and 3 m^2^ m^−2^. We chose these four levels to represent mild canopy shading (LAI_C = 0.5 and 1 m^2^ m^−2^) and heavy canopy shading (LAI_C = 2 and 3 m^2^ m^−2^). The reduction of PAR by each level of LAI_C was calculated according to the Beer–Lambert equation using a value of 0.6 for the light extinction coefficient (Yin and Struik, 2015) (Supplementary Sata Fig. S6). Note that by using the Beer–Lambert equation, we embedded an assumption in our simulations that the overhead canopy was homogeneous. The reduction of R:FR by the overhead canopy was calculated based on the fraction of reduced PAR according to eqn (4) (Evers *et al*., 2006) (Fig. S6):

$$\begin{array}{l} \;\;\;R:FR=R:FR_{non\text{\textasciitilde{}}\text{-}shading}\times exp(-2.32\times f_{PAR\;\;\;intercepted}) \end{array}$$

where R:FR_non-shading_ is the incoming R:FR under non-shaded conditions (=1.05) and *f*_PAR intercepted_ is the fraction of PAR intercepted by the canopy.

Virtual plant phenotypes under each level of canopy shading were created based on the assumption that plant parameters were linearly related to the PAR and R:FR levels. The linear relationships between plant parameters and the PAR and R:FR levels were quantified using the values obtained in the experiment (Supplementary Data Fig. S7). By changing one plant parameter value or values of a set of parameters at a time, we estimated the effect of individual trait responses on the fraction of light interception and plant photosynthesis using eqn (5):

$$\begin{array}{l} E=(Y_{plastic}-Y)/Y \end{array}$$

where *E* is the relative effect of trait responses on the fraction of light interception or plant photosynthesis; *Y*_plastic_ is either the fraction of light interception or plant photosynthesis that was calculated under the reduced incoming light intensity by LAI_C, using the target plastic trait values that are changed according to the PAR and R:FR levels under LAI_C, while keeping all other trait values the same as values of the non-shaded plant phenotype; and *Y* is either the fraction of light interception or plant photosynthesis that was calculated under the reduced incoming light intensity by LAI_C, using the non-shaded plant phenotype.

The interaction between trait effects on plant photosynthesis was estimated using eqn (6):

$$\begin{array}{l} I=E_{total}-E_{additive} \end{array}$$

where *E*_total_ is the *E* (in eqn 5) of which *Y*_plastic_ is calculated by changing all target plastic traits simultaneously, and *E*_additive_ is the sum of *E* values estimated for each individual target trait.

## RESULTS

### Experimental effects of PAR and R:FR on plant architectural and leaf photosynthetic traits

Plant height, total leaf area, number of leaves and individual leaf area decreased with a reduction in PAR, but PAR did not significantly affect internode length or leaf inclination angle (Table 2, Supplementary Data Table S3). The maximum leaf photosynthetic rate (*A*_max_) decreased by reduced PAR, while PAR did not significantly affect other leaf photosynthetic parameters (dark respiration rate *R*_d_, quantum efficiency *Φ*_CO2LL(inc)_ and the curvature factor of the light response curve *θ*) (Table 3). The slope *s* of the linearly correlation between *A*_max_ and *R*_d_ decreased with lower PAR (Table 3).

**Table 2.** Measured effects of reductions in photosynthetically active radiation (PAR) and red to far-red ratio (R:FR) on architectural traits at plant level and organ level (an example of internode and leaf at rank 6)

Treatment		Plant level			Organ level (rank 6)		
PAR	R:FR	Plant height (cm)	Total leaf area (cm^2^)	Number of leaves	Internode length (cm)	Leaf area (cm^2^)	Leaf inclination angle (°)
High	High	62.6 ± 0.8 ab	778 ± 48 a	12 ± 0.4 a	5.0 ± 0.2 bc	87 ± 7 ab	29 ± 4 b
High	Medium	66.7 ± 3.5 a	781 ± 92 a	12 ± 0.2 ab	5.4 ± 0.1 a	88 ± 7 a	38 ± 7 ab
High	Low	66.0 ± 2.3 a	740 ± 43 a	11 ± 0.6 cd	5.3 ± 0.4 ab	90 ± 1 a	40 ± 6 a
Medium	High	60.0 ± 2.9 b	714 ± 15 a	11 ± 0.4 abc	5.0 ± 0.1 abc	77 ± 1 c	33 ± 10 ab
Medium	Low	62.4 ± 5.6 ab	724 ± 26 a	11 ± 0.4 d	5.3 ± 0.3 ab	81 ± 2 bc	40 ± 4 a
Low	High	52.9 ± 4.0 c	629 ± 48 b	11 ± 0.2 bcd	4.7 ± 0.3 c	70 ± 3 d	32 ± 7 ab

Values are mean ± s.d. from four statistical replicates, each of which includes five plants. Letters following the numbers in each column indicate significant differences when comparing between treatments (*P* < 0.05).

**Table 3.** Leaf photosynthetic parameters estimated for leaves at upper, middle and lower level of the plant

Treatment	PAR R:FR	High High	High Low	Medium High	Medium Low
*A*_max_ (*μ*mol m^−2^ s^−1^)					
Upper leaf		16.1 ± 1.5 a	17.2 ± 2.2 a	11.7 ± 1.2 b	12.2 ± 0.6 b
Middle leaf		14.0 ± 2.3 a	16.1 ± 2.4 a	10.8 ± 1.7 b	10.0 ± 1.2 b
Lower leaf		10.7 ± 2.2 a	10.0 ± 1.0 ab	7.9 ± 1.1 b	8.2 ± 1.5 ab
*R*_d_ (*μ*mol m^−2^ s^−1^)					
Upper leaf		0.85 ± 0.39 a	0.83 ± 0.39 a	0.25 ± 0.08 b	0.48 ± 0.17 ab
Middle leaf		0.53 ± 0.38 a	0.77 ± 0.28 a	0.50 ± 0.43 a	0.25 ± 0.18 a
Lower leaf		0.45 ± 0.18 a	0.35 ± 0.18 a	0.19 ± 0.33 a	0.28 ± 0.08 a
*Φ*_CO2LL(inc)_ (mol CO_2_ mol^−1^ photon)					
Upper leaf		0.051 ± 0.006 a	0.053 ± 0.010 a	0.046 ± 0.006 a	0.048 ± 0.003 a
Middle leaf		0.052 ± 0.007 a	0.056 ± 0.007 a	0.053 ± 0.005 a	0.052 ± 0.009 a
Lower leaf		0.048 ± 0.007 a	0.047 ± 0.007 a	0.041 ± 0.008 a	0.045 ± 0.005 a
*θ*					
Upper leaf		0.70 ± 0.05 a	0.47 ± 0.21 b	0.70 ± 0.05 a	0.59 ± 0.01 ab
Middle leaf		0.71 ± 0.07 ab	0.56 ± 0.1 c	0.76 ± 0.04 a	0.61 ± 0.09 bc
Lower leaf		0.71 ± 0.06 a	0.46 ± 0.11 b	0.72 ± 0.07 a	0.58 ± 0.13 ab
Slope *s*		0.05 ± 0.01 a	0.05 ± 0.01 a	0.03 ± 0.01 b	0.03 ± 0.01 b
Coefficient *k*		0.12 ± 0.02 b	0.15 ± 0.03 ab	0.13 ± 0.03 ab	0.19 ± 0.04 a

*A*_max_ is the maximum leaf photosynthetic rate at saturating incident light. *R*_d_ is the dark respiration rate. *Φ*_CO2LL(inc)_ is the quantum yield of CO_2_ assimilation on the basis of incident light. *θ* is the curvature factor of the light response curve. *s* is the slope of the linear regression between *A*_max_ and *R*_d_. *k* is the coefficient describing the relationship between light gradient and *A*_max_ gradient in the canopy. Values of *A*_max_, *R*_d_, *Φ*_CO2LL(inc)_ and *θ* are mean ± s.d. from four statistical replicates, each of which includes two plants. Values of *s* and *k* are estimate ± error of estimation. Letters following the numbers in each row indicate significant differences when comparing between treatments (*P* < 0.05).

Internode length and leaf inclination angle increased under reduced R:FR, but R:FR did not significantly affect other plant architectural traits (Table 2, Supplementary Data Table S3). *θ* decreased with reduced R:FR, while R:FR did not significantly affect *A*_max_, *R*_d_ or *Φ*_CO2LL(inc)_ (Table 3). Reductions in R:FR tended to increase the coefficient *k* (describing the relationship between light gradient and *A*_max_ gradient in the canopy) (Table 3), indicating that the decline in *A*_max_ from more illuminated leaves in the top of the canopy to the more shaded ones lower down might be steeper in low R:FR plants than in high R:FR plants.

### Evaluation of the FSP model of rose

**Fig. 1.**
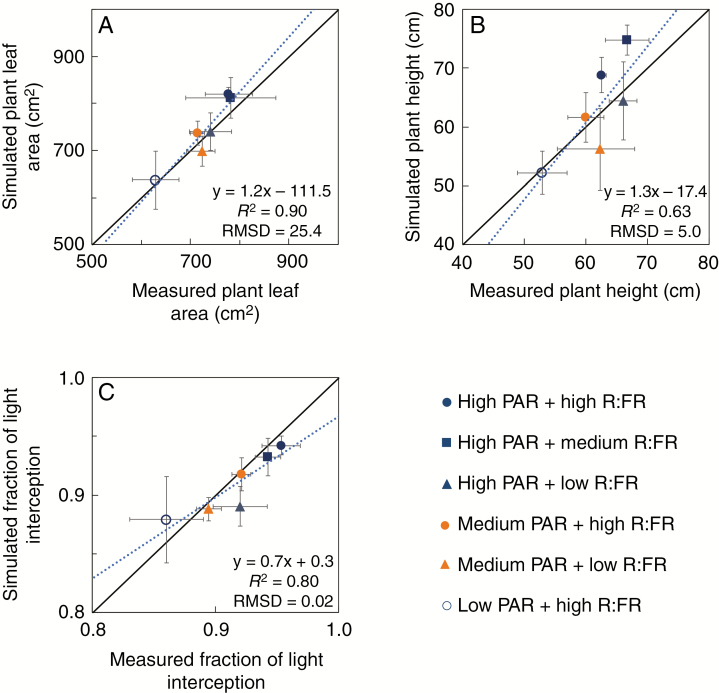
Comparisons between measured and simulated results of (A) plant total leaf area, (B) plant height and (C) the fraction of light intercepted by the plants in different photosynthetically active radiation (PAR) and red to far-red ratio (R:FR) treatments. The equation in each panel represents the linear regression of simulated (*y*) vs. measured (*x*) values. *R*^2^ is the determination coefficient of the linear regression. RMSD is the relative root-mean-square deviation. Solid lines are 1:1 lines. Dotted lines are linear regression lines. Error bars are standard deviations.

**Fig. 2.**
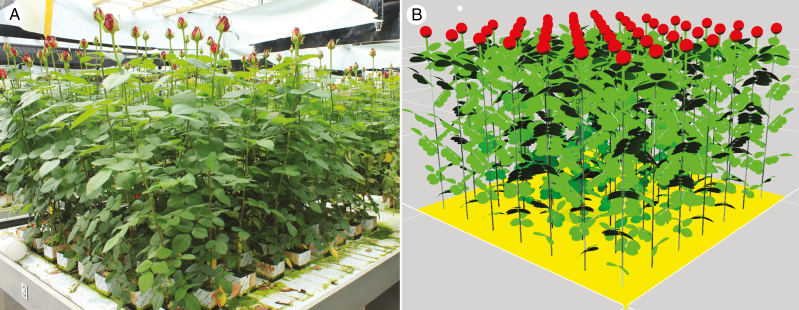
Comparison of (A) a rose plant population under treatment with high photosynthetically active radiation and high red to far-red ratio in the experiment with (B) a simulated rose population without canopy shading at the flowering stage.

As the FSP model was built based on direct measurements of organ size, the model was validated using data collected at the plant and canopy levels. Plant total leaf areas, plant heights and the fraction of light intercepted by the plants simulated with our model for different treatments closely matched measured values (Fig. 1). *r*^2^ and RMSD were respectively 0.90 and 25.4 for plant leaf area, 0.63 and 5.0 for plant height, and 0.80 and 0.02 for the fraction of light interception. However, a few data points had relatively large standard deviations (e.g. the fraction of light interception of the low PAR and high R:FR treatment in Fig. 1C), indicating a fair amount of scatter in the results of individual replicates. The comparison of virtual plants and real plants showed that the 3D architecture of virtual plants simulated by the model was similar to that of real plants in the experiment (Fig. 2).

### Simulated separate effects of responses to PAR and R:FR on plant light interception and photosynthesis

**Fig. 3.**
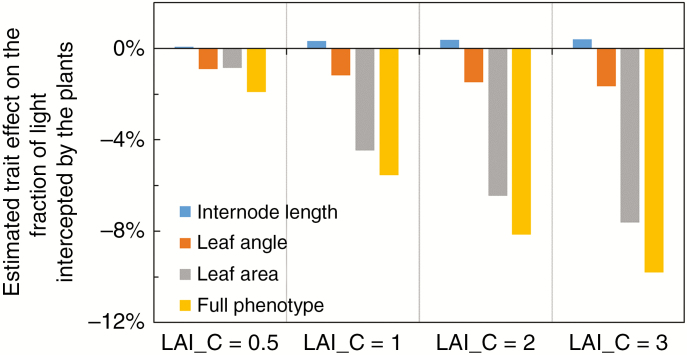
Estimated effects of plant architectural trait responses to reduced photosynthetically active radiation (PAR) (i.e. leaf area), to reduced red to far-red ratio (R:FR) (i.e. internode length and leaf angle), and to combination of the two (i.e. ‘Full phenotype’) on the fraction of light intercepted by the plants under canopy shading caused by a leaf area index (LAI_C) of 0.5, 1, 2 and 3 m^2^ m^−2^. Trait effects are calculated as the relative changes of light intercepted by plants caused by changing targeted traits compared with light intercepted by the non-shaded plant phenotype (see eqn 5 for the calculation). ‘Full phenotype’ represents the phenotype that trait responses to both reduced PAR and reduced R:FR are changed simultaneously.

Simulation results showed that plant architectural responses to canopy shading as a whole (i.e. introducing the combined effects of R:FR and PAR on internode length, leaf angle and leaf area) decreased the fraction of light interception, and this negative effect increased from −2 % to −10 % when shading LAI (LAI_C) increased from 0.5 to 3 m^2^ m^−2^ (Fig. 3). Decreased leaf area as a result of reductions in PAR had the largest effect on the fraction of light interception (−1 % to −8 %) (Fig. 3). Increased leaf inclination angle due to reductions in R:FR had a minor effect on the fraction of light interception (−1 % to −2 %) (Fig. 3). Increased internode length had little effect on the fraction of light interception (Fig. 3).

**Fig. 4.**
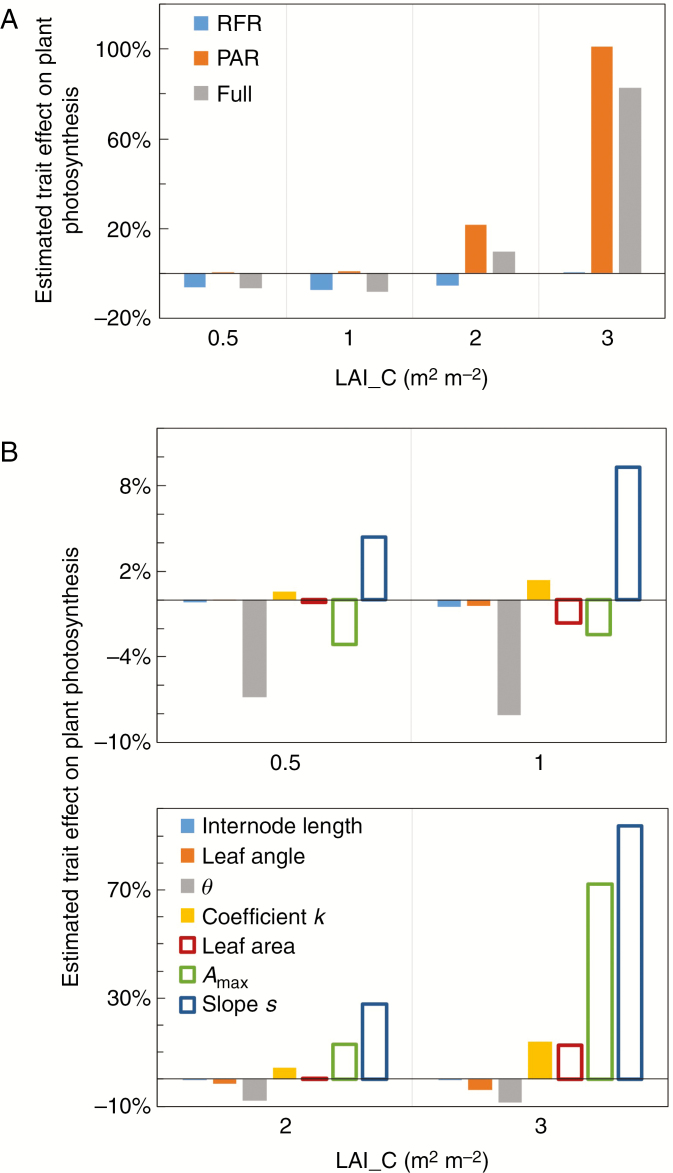
Estimated effects of (A) the combination of traits and (B) individual traits on plant photosynthesis under canopy shading caused by a leaf area index (LAI_C) of 0.5, 1, 2 and 3 m^2^ m^−2^. Trait effects are calculated as the relative changes of plant photosynthesis by changing targeted traits compared with plant photosynthesis of non-shaded plant phenotype (see eqn 5 for the calculation). In A, ‘RFR phenotype’ represents the phenotype that all trait responses to reduced red to far-red ratio (R:FR) are changed simultaneously, ‘PAR phenotype’ represents the phenotype that all trait responses to reduced photosynthetically active radiation (PAR) are changed simultaneously, and ‘Full phenotype’ represents the phenotype that all trait responses to both reduced R:FR and reduced PAR are changed simultaneously. In B, solid bars are trait responses to reduced R:FR, while open bars are trait responses to reduced PAR.

Plant responses to canopy shading as a whole (i.e. ‘Full phenotype’; the combination of all trait responses to both reduced PAR and reduced R:FR) decreased plant net photosynthesis by 7–8 % at mild shade levels (LAI_C = 0.5 and 1 m^2^ m^−2^) but increased net photosynthesis by 10–83 % at heavy shade levels (LAI_C = 2 and 3 m^2^ m^−2^) (Fig. 4A). The relative contribution to these effects by plant responses to the two shading factors (i.e. reduced PAR and reduced R:FR) tended to be in the opposite direction and depended on the level of shading. At mild shade levels, the reduction in photosynthesis was mainly caused by plant responses to reduced R:FR, whereas at heavy shade levels, the increase in photosynthesis was mainly caused by responses to reduced PAR (Fig. 4A).

Among all trait responses to reduced PAR, changes in slope *s* had the largest effects on plant photosynthesis (4–94 %) at all shade levels, followed by changes in *A*_max_ (−3 to 72 %; note that the effect of *A*_max_ was both direct through changes in *A*_max_ itself and indirect through changes in *R*_d_, see eqn 2) and leaf area (−2 to 13 %). With increasing level of shading, all trait responses to reduced PAR tended to more positively affect photosynthesis (Fig. 4B). Among all trait responses to reduced R:FR, the relative effect of changes in the coefficient *k* on plant photosynthesis increased (1–14 %) while the relative effect of changes in *θ* became more negative (−7 to −9 %) with increasing level of shade (Fig. 4B). Changes in internode length had an effect of <1 % on plant photosynthesis (Fig. 4B). Changes in leaf inclination angle had little effect on plant photosynthesis under mild shade (an effect < 1 %), but decreased plant photosynthesis by 2–4 % under heavy shade (Fig. 4B).

### Simulated interactive effects of responses to PAR and R:FR on plant photosynthesis

**Fig. 5.**
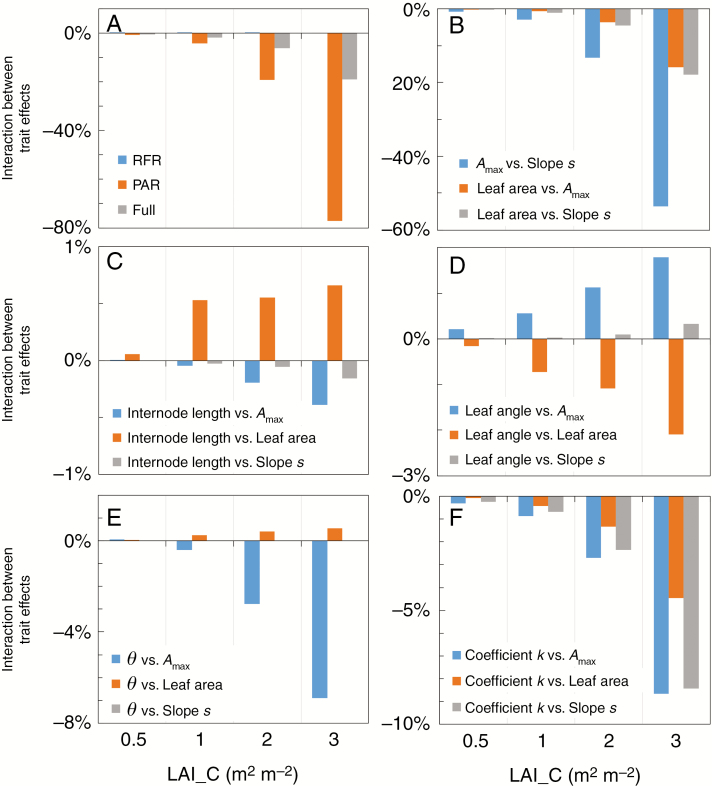
Estimated interaction between effects of trait responses on plant photosynthesis under canopy shading caused by a leaf area index (LAI_C) of 0.5, 1, 2 and 3 m^2^ m^−2^. Interactions of trait effects are calculated as the differences between the combined effects of changing all targeted traits on plant photosynthesis and the additive effects of separately changing individual targeted traits on plant photosynthesis (see eqn 6 for the calculation). In A, ‘RFR’ represents the interaction between effects of all trait responses to reduced red to far-red ratio (R:FR), ‘PAR’ represents the interaction between effects of all trait responses to reduced photosynthetically active radiation (PAR), and ‘Full’ represents the interaction between effects of all trait combinations to reduced R:FR and all trait combinations to reduced PAR. In B, each bar represents the interaction between the effects of two individual low-PAR responses. In C–F, each bar represents the interaction between the effect of an individual low-R:FR response and the effect of an individual low-PAR response.

Simulation results showed that the effects of individual trait responses to reduced PAR on plant photosynthesis negatively interacted with each other (Fig. 5A), in that effects of combined trait responses on plant photosynthesis were less positive than when effects of individual trait responses were added. The largest negative interaction occurred between the effect of changing *A*_max_ and the effect of changing the slope *s* on plant photosynthesis (Fig. 5B). Such a negative interaction indicates that the effect of lowering *R*_d_ by reducing *s* on plant photosynthesis became less when *R*_d_ had already been reduced by lowering *A*_max_, in that the effect of simultaneous changes in *A*_max_ and *s* on plant photosynthesis was less positive than when the effects of separately changing *A*_max_ and *s* on plant photosynthesis were added. Conversely, there was hardly any interaction between effects of trait responses to reduced R:FR (Fig. 5A). Further simulation studies showed that interactions between each low-R:FR response were very small (<1 %) and occurred in both positive and negative directions (Supplementary Data Fig. S8), leading to compensations among each other, and thus no overall interaction between the effects of individual low-R:FR responses.

The negative interaction also existed between the effect of ‘PAR phenotype’ (i.e. the combination of all trait responses to reduced PAR) and the effect of ‘RFR phenotype’ (i.e. the combination of all trait responses to reduced R:FR) on plant photosynthesis (Fig. 5A). This indicates that the effects of ‘Full phenotype’ on plant photosynthesis were more negative under mild shade levels and were less positive under heavy shade levels than when the effects of ‘PAR phenotype’ and ‘RFR phenotype’ were added (as also shown in Fig. 4A). The negative interaction between the effects of ‘PAR phenotype’ and ‘RFR phenotype’ on plant photosynthesis was mainly caused by the interaction between *θ* and *A*_max_ (i.e. the effect of changing *A*_max_ is smaller when *θ* is reduced; Fig. 5E) and the interaction between coefficient *k* and all low-PAR responses (changes in *A*_max_, leaf area and *s*) (i.e. the effect of changing the distribution of *A*_max_ in the canopy is smaller when *A*_max_ itself is lower, when leaf area is reduced, and when the slope *s* for the linear correlation of *A*_max_ and *R*_d_ is lower; Fig. 5F). Some trait effects (e.g. those of internode length and leaf area) interacted positively with each other, but these interactive effects were small (<2 %, Fig. 5C–E).

## DISCUSSION

### The relative importance of individual shade responses for plant photosynthesis changes with shade level

**Fig. 6.**
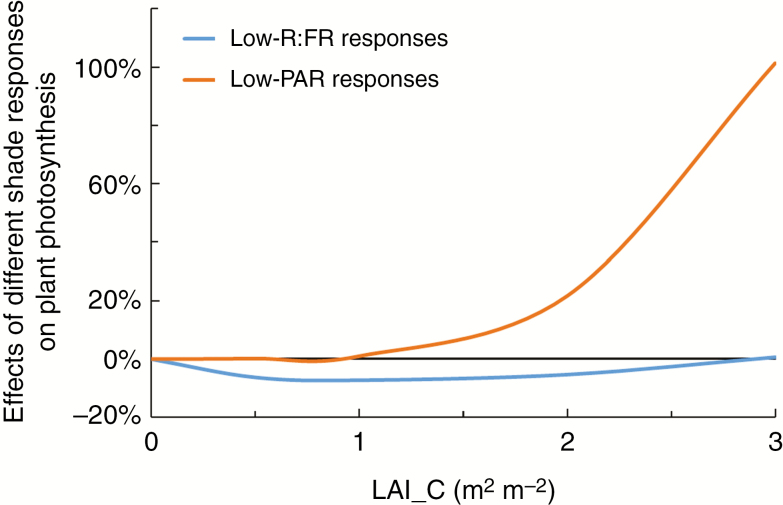
Concept figure of changing the dominant factor that affects plant photosynthesis with increasing level of canopy shading (LAI_C).

Understanding the adaptive significance of plastic responses to canopy shading involves quantifying how responses to the individual shading factors interact in determining plant functions such as plant photosynthesis. Here, we have shown that plastic responses to reduced PAR and reduced R:FR involve different traits, and that effects of plastic responses to these two shading factors on plant photosynthesis are different and can operate in opposite directions. In addition, the directions of individual trait effects and their relative importance changed with the level of canopy shading, with effects of responses to low R:FR being more dominant at mild shading and effects of responses to low PAR dominating at heavy shading (Figs 4A and 6). This is in line with the common view that reductions in R:FR operate as an early warning signal for future shading, in contrast to decreases in PAR which occur only under shading (Ballaré, 1999).

In the early stages of canopy development, plants could immediately experience significant reductions in R:FR when reductions in PAR are still absent or relatively minor (Ballaré, 1999). Therefore, low R:FR is widely considered as an early warning signal for plants regarding the proximity of neighbours, and the subsequent shade avoidance responses are considered to improve plant performance by preventing plants from becoming shaded (Ballaré, 1999; Vandenbussche *et al*., 2005; López Pereira *et al*., 2017). Simulations in this study also showed that at mild shade levels, responses to reductions in R:FR were more important for plant performance, as the effects of low-R:FR responses on plant photosynthesis were larger than the effects of low-PAR responses (Figs 4A and 6). However, in our simulations in which constant shade was considered, the effects of low-R:FR responses on plant photosynthesis were negative (Fig. 4A). This indicates that when shade avoidance responses induced by low R:FR do not eventually lead to greater light acquisition, by improving access to light, these responses are not beneficial to plant photosynthesis. Moreover, leaf physiological changes induced by low R:FR (i.e. changes in the curvature of light response curves and the correlation between light gradient and *A*_max_ gradient in the canopy) had a relatively large impact on plant photosynthesis compared to typically observed shade avoidance responses in plant architecture (i.e. internode elongation and leaf hyponasty) (Fig. 4B). These results suggest that apart from the well-observed architectural responses to reduced R:FR, leaf physiological responses to reduced R:FR also substantially affect plant performance.

With the canopy fully developing, plants experience continuing reductions in PAR while the R:FR ratios stay at constantly low levels (Supplementary Data Fig. S6). Simulations showed that under heavy shading, responses to low PAR were more important for plant photosynthesis than responses to low R:FR (Fig. 4A), indicating that plasticity to low PAR is high under heavy shading. Plant responses to low PAR help to maintain a positive carbon balance under canopy shading, as our simulations showed positive effects of responses to low PAR on plant photosynthesis (Fig. 4A). The positive effect of trait responses to low PAR on plant photosynthesis was mainly caused by responses that reduce respiration costs (Fig. 4B). Walters & Reich (2000) also showed that under low light conditions, minimizing carbon loss is more beneficial to plants than maximizing carbon gain. These results suggest that under canopy shading, plant plasticity to low PAR is beneficial to plant carbon balance due to reduced respiration costs.

In our simulations, lower *R*_d_ was associated with decreases in both *A*_max_ and the slope *s* of the linear relationship between *A*_max_ and *R*_d_ (Fig. 4B). Under low light, both *A*_max_ and *R*_d_ have been found to decrease (Sims and Pearcy, 1991; Walters *et al*., 1993), as also found in our experiment (Table 3). Furthermore, *R*_d_ has been found to decrease more strongly than *A*_max_ (Sims and Pearcy, 1991), as also suggested by our result that the slope *s* decreased with reductions in PAR (Table 3). Although the lower leaf area and lower *A*_max_ had little effect on net plant photosynthesis at mild shading, reductions in leaf area and *A*_max_ positively affected plant photosynthesis under heavy shading (Fig. 4B). Such a positive effect is relevant with the associated reduction in respiration, as our simulations showed that if *A*_max_ and *R*_d_ were not correlated, decreasing *A*_max_ would reduce plant photosynthesis (Supplementary Data Fig. S9).

### Effects of low-R:FR responses and low-PAR responses on plant photosynthesis negatively interact

Our simulations showed a negative interaction between effects of low-R:FR responses and effects of low-PAR responses on plant photosynthesis (Fig. 5A). This negative interaction was mainly caused by interactions between the effect of changing coefficient *k* induced by low R:FR and effects of individual low-PAR responses (Fig. 5F). A higher *k* value indicates a steeper *A*_max_ gradient in the canopy. Such a steeper decline in *A*_max_ means that the heavy shaded leaves lower in the canopy have less photosynthetic capacity, resulting in less respiration costs in these leaves (Supplementary Data Fig. S10A,B). This could potentially improve photosynthesis of the whole plant especially at heavy shading when leaves in the lower canopy receive hardly any light resource for photosynthesis and these leaves are mainly consuming rather than producing assimilates (Fig. S10C). However, when *A*_max_ itself decreases, the more illuminated leaves in the upper canopy also have a lower capacity for photosynthesis, which mitigates the earlier mentioned positive effect of increasing *k* value on plant photosynthesis (Fig. 5F).

More generally, these results show that effects of low-PAR responses on plant photosynthesis depend on effects of low-R:FR responses and vice versa. This suggests that if there is genetic variation in plant plasticity to different shading factors (reduced PAR and reduced R:FR), selection for plasticity to one shading factor depends on the level of plasticity to the other factor. This result further connects to the broader literature on divergent evolution of shade responses, which has shown differentiation in plasticity to low R:FR between ecotypes from different shade habitats. Typically ecotypes (e.g. in the annual *Impatiens capensis*) from forest habitats experiencing shading from taller plants show much reduced low-R:FR responses compared with grassland ecotypes experiencing shading from more similar sized plants (Dudley and Schmitt, 1995; Donohue and Schmitt, 1999; Donohue *et al*., 2000; Huber *et al*., 2004; Anten *et al*., 2009). The question arising from our work is whether this divergence also involves a different balance between responses to low R:FR and low PAR.

### Limitations of this study and future perspectives

Essentially, our simulations mimic the situation whereby target plants are shaded by an overhead canopy. This situation is commonly found in the forest understorey, or in certain intercropping and agro-forestry systems. However, as noted, plants also often experience canopy shading caused by crowding of similar-sized neighbours (e.g. high plant density), which, while not necessarily taller than target plants, still cause reductions in R:FR and the amount of PAR available for individual plants. In those situations, responses to low R:FR may be relatively more important than those to low PAR. We did not simulate those situations because our experiment did not allow us to make reasonable assumptions to create reliable virtual phenotypes in crowding populations. However, if combined with appropriate experiments, the modelling approach presented in our study could account for those situations too.

We quantified the effects of all trait responses to reduced PAR and R:FR under canopy shading and compared their relative importance by using a combination of experimentation and modelling. Next to the two shading factors (low PAR and low R:FR) investigated in this study, the intensities of blue light and green light are changed by canopy shading as well. The reduction of blue light is known to induce shade avoidance responses in plants (reviewed by Keuskamp *et al*., 2012). Increases in the fraction of green light have also been suggested to induce shade avoidance responses (Zhang *et al*., 2011). Therefore, plant responses to other shading factors may potentially interact with plant responses to low PAR and low R:FR. In addition, other environmental changes may involve multiple factors similar to canopy shading; for example, wind involves both mechanical stress and micro-climatic changes (Anten *et al*., 2010). A combination of experiments that can separate individual environmental factors and FSP modelling is a useful tool to disentangle the effects of individual plastic architectural and physiological trait responses to these factors on plant performance and to investigate the interactions between trait effects.

## CONCLUSIONS

Shading by an overhead canopy entails reductions in both PAR and R:FR, which induce responses in leaf photosynthetic traits and plant architectural traits that can affect plant photosynthesis. Using a 3D plant model, we disentangled these responses and showed that effects of these responses on plant photosynthesis can operate in opposite directions and can be strongly inter-dependent. The relative importance of these responses on plant photosynthesis also changes with the level of canopy shade. Our study indicates that environmental changes entail multiple factors that induce responses in different plant traits, which can be separately studied using model simulations. Our simulation results indicate that the effect of a response in one trait on plant performance depends strongly on the response in other traits.

## SUPPLEMENTARY DATA

Supplementary data are available online at https://academic.oup.com/aob and consist of the following.

Figure S1: measurements of light spectrum at wavelength of 400–800 nm.

Figure S2: climate conditions inside the glasshouse during the experiment.

Figure S3: the relationship between light gradient and gradient of leaf photosynthetic capacity in the canopy.

Figure S4: measurements of internode length and leaf area at each phytomer rank, and the comparison between measured and calculated internode length and leaf area.

Figure S5: measurements of leaflet number at each leaf rank.

Figure S6: reductions of PAR and R:FR in response to the increasing level of canopy shading.

Figure S7: the relationship between changes in R:FR or PAR with changes in plant architectural traits.

Figure S8: estimated interactions between effects of individual trait responses to low R:FR on plant photosynthesis under canopy shading.

Figure S9: estimated effect of plasticity in leaf photosynthetic capacity on plant photosynthesis, with no correlation between changing *A*_max_ and the dark respiration rate under canopy shading.

Figure S10: the effect of changing the correlation between light gradient and *A*_max_ gradient in the canopy on *A*_max_, *R*_d_ and net leaf photosynthetic rate of individual leaves on the plant.

Table S1: measured R:FR under different numbers of additional far-red lamps with assimilation lighting on or off.

Table S2: leaf spectrophotometric measurements.

Table S3: measured effects of reductions in PAR and R:FR on plant architectural traits.

Method S1: estimation of leaf photosynthetic parameters.

mcz197_suppl_Supplementary_Figures

## FUNDING

We are grateful for financial support from the China Scholarship Council (CSC) (No. 201406850003) and from the project ‘More roses for less’ (No. 870.15.040) funded by the Netherlands Organisation for Scientific Research (NWO), Signify and Glastuinbouw Nederland.
