# Supplementary material for: Disentangling the effects of photosynthetically active radiation and red to far-red ratio on plant photosynthesis under canopy shading: a simulation study using a functional–structural plant model
Source: Ann Bot. 2019 Dec 3;126(4):635–46. doi: 10.1093/aob/mcz197 (PMC7489061; doi:10.1093/aob/mcz197)
Supplement: mcz197_suppl_Supplementary_Figures [file mcz197_suppl_supplementary_figures.docx]

Fig. S1. Measurements of light spectrum at wavelength 400-800 nm for treatment ‘high photosynthetically active radiation (PAR) + high red to far-red ratio (R:FR)’ (A), ‘high PAR + low R:FR’ (B), ‘medium PAR + high R:FR’ (C) and ‘medium PAR + low R:FR’ (D). Relative light intensity is the light intensity measured for each wavelength divided by the total light intensity. Four far-red lamps were added in treatments which had low R:FR. One layer of neutral shading screen was used in treatments which had medium PAR.

Fig. S2. Climate conditions inside the greenhouse during the experiment. (A) Daily average photosynthetically active radiation (PAR) level during the light period. The PAR level included both natural sunlight and the assimilation lighting if it was on. (B) Daily average relative humidity. (C) Average day and night temperature. Each symbol (● or ×) represents an average value that calculated based on the climate data that collected every five minutes.

Fig. S3. The relationship between light gradient and the gradient of leaf photosynthetic capacity in the canopy. *Q*_0_ / *Q*_top_ is the relative light intensity, calculated as light intensity measured at middle or low level of the plants (*Q*_0_) divided by the light intensity measured at top of the plants (*Q*_0_). *A*_max,0_ is the leaf photosynthetic capacity estimated for leaves at middle or low level of the plants. *A*_max,top_ is the leaf photosynthetic capacity estimated for the top leaf of the plants. Error bars are standard deviations.

Fig. S4. Measurements of (A) internode length and (B) leaf area at each phytomer rank on the plant, and comparisons between the measured and calculated (C) internode length and (D) leaf area, assuming that internode length or leaf area between every second phytomer can be linear interpolated. In panels (A) and (B), dots and error bars are respectively means and standard deviations of three replicates, with each replicate consisting of six individual plants. In panels (C) and (D), dots are internode length or leaf area of one phytomer on an individual plant, and rRMSE is the relative root-mean-square error.

Fig. S5. Leaflet number of the compound leaf at each rank of the plant measured in each treatment with different levels of photosynthetically active radiation (PAR) and red to far-red ratio (R:FR). Leaf rank is counted from the bottom of the plant towards the flower bud. Error bars are standard deviations.


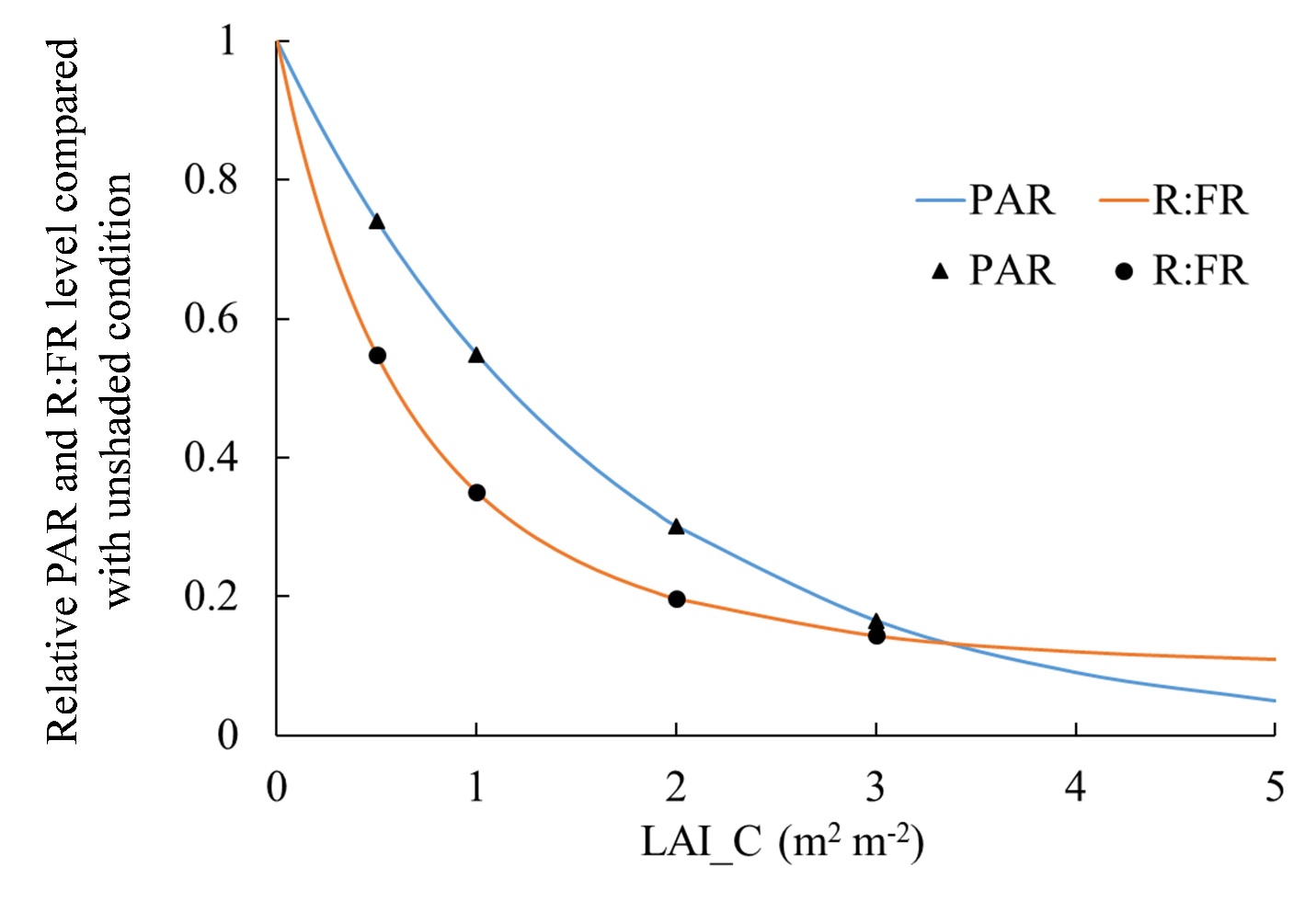


Fig. S6. The relationship between the relative level of photosynthetically active radiation (PAR) and red to far-red ratio (R:FR) compared with the non-shaded condition and the leaf area index of the overhead canopy (LAI_C). Lines are simulated results. Triangles and circles are the relative PAR and R:FR levels at LAI_C = 0.5, 1, 2 and 3 m^2^ m^-2^.

Fig. S7. The relationship between (A) changes in red to far-red ratio (R:FR) and internode length, (B) changes in R:FR and leaf inclination angle, and (C) changes in photosynthetically active radiation (PAR) and leaf area. Error bars are standard deviations. Equation following the linear regression line for each rank is the regression equation for the organ trait at that rank.

Fig. S8. Estimated interactions between effects of individual trait responses to low red to far-red ratio on plant photosynthesis under canopy shading caused by a leaf area index (LAI_C) of 0.5, 1, 2 and 3 m^2^ m^-2^. Each bar represents the interaction between two individual traits. ‘Theta’ is the curvature factor *θ* of light response curve; coefficient *k* describes the correlation between light gradient and the gradient of leaf photosynthetic capacity in the canopy.

**
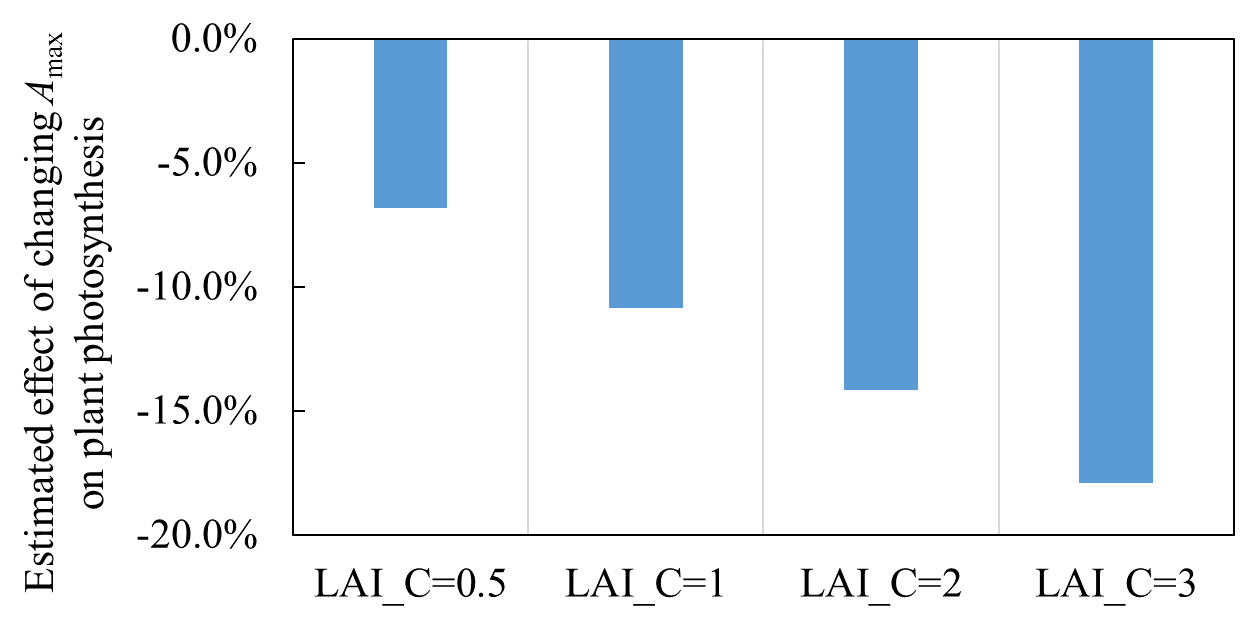
**

Fig. S9. Estimated effect of plasticity in leaf photosynthetic capacity (*A*_max_) on plant photosynthesis, with no correlation between changing *A*_max_ and the dark respiration rate, at shade levels caused by a leaf area index (LAI_C) of 0.5, 1, 2 and 3 m^2^ m^-2^.

Fig. S10. Simulations of (A) leaf photosynthetic capacity, (B) respiration rate and (C) net leaf photosynthetic rate of individual leaves on the plant under shading by a canopy with leaf area index of 3 m^2^ m^-2^. The background light intensity was set at 500* *mol m^-2^ s^-1^, with a 73% reduction by the shading canopy, resulting in the actual incoming light intensity of 85 *μ*mol m^-2^ s^-1^. The coefficient *k* describes the relationship between light gradient and the gradient of leaf photosynthetic capacity in the canopy. *k* value of 0.12 and 0.17 respectively represents the parameter value of the non-shaded plant phenotype and the plant phenotype obtained under canopy shading.

Table S1.Measured red to far-red ratio (R:FR) in treatments in which had 0, 2 or 4 additional far-red lamps above the canopy at the conditions when assimilation lighting were on or off. R:FR values are mean ± SD (obtained from nine measurement points).

| Number of additional far-red lamps above the canopy | The use of assimilation lighting | Measured R:FR |
| --- | --- | --- |
| 0 | No | 1.04 ± 0.02 |
| 0 | Yes | 1.24 ± 0.08 |
| 2 | No | 0.29 ± 0.06 |
| 2 | Yes | 0.48 ± 0.07 |
| 4 | No | 0.26 ± 0.03 |
| 4 | Yes | 0.27 ± 0.06 |

Table S2.Leaf reflectance and transmittance of photosynthetically active radiation (PAR, 400-700 nm) measured in each treatment. Leaf PAR absorbance was calculated based on reflectance and transmittance. Values are mean ± SD, which are obtained from six leaf discs. Average transmittance, reflectance and absorbance values of two leaf sides were used in the rose model.

| Treatment | PAR  R:FR | High  High | High  Medium | High  Low | Medium  High | Medium  Low | Low  High |
| --- | --- | --- | --- | --- | --- | --- | --- |
| Leaf adaxial side | | | | | | | |
| Transmittance | | 0.05 ± 0.00 | 0.04 ± 0.01 | 0.05 ± 0.01 | 0.06 ± 0.01 | 0.06 ± 0.00 | 0.06 ± 0.01 |
| Reflectance | | 0.05 ± 0.00 | 0.05 ± 0.00 | 0.05 ± 0.00 | 0.05 ± 0.00 | 0.05 ± 0.00 | 0.05 ± 0.00 |
| Absorbance | | 0.90 ± 0.00 | 0.91 ± 0.01 | 0.90 ± 0.01 | 0.89 ± 0.01 | 0.89 ± 0.00 | 0.89 ± 0.01 |
| Leaf abaxial side | | | | | | | |
| Transmittance | | 0.07 ± 0.01 | 0.05 ± 0.00 | 0.05 ± 0.01 | 0.09 ± 0.01 | 0.08 ± 0.01 | 0.09 ± 0.00 |
| Reflectance | | 0.11 ± 0.01 | 0.12 ± 0.00 | 0.10 ± 0.02 | 0.11 ± 0.01 | 0.10 ± 0.00 | 0.11 ± 0.01 |
| Absorbance | | 0.82 ± 0.02 | 0.84 ± 0.00 | 0.85 ± 0.03 | 0.80 ± 0.01 | 0.82 ± 0.01 | 0.81 ± 0.01 |
| Average of two leaf sides | | | | | | | |
| Transmittance | | 0.06 ± 0.01 | 0.05 ± 0.00 | 0.05 ± 0.01 | 0.07 ± 0.01 | 0.07 ± 0.00 | 0.07 ± 0.00 |
| Reflectance | | 0.08 ± 0.00 | 0.08 ± 0.00 | 0.08 ± 0.01 | 0.08 ± 0.00 | 0.07 ± 0.00 | 0.08 ± 0.00 |
| Absorbance | | 0.86 ± 0.01 | 0.87 ± 0.00 | 0.87 ± 0.02 | 0.85 ± 0.01 | 0.86 ± 0.00 | 0.85 ± 0.00 |

Table S3.Measured effects of reductions in photosynthetically active radiation (PAR) and red to far-red ratio (R:FR) on internode length, leaf area and leaf inclination angle at rank 2, 4, 8 and 10. Values are mean ± SD from four statistical replicates, each of which includes five plants. Letters following the numbers in each row indicate significant differences when comparing between treatments (*P* < 0.05).

| Treatment | PAR  R:FR | High  High | High  Medium | High  Low | Medium  High | Medium  Low | Low  High |
| --- | --- | --- | --- | --- | --- | --- | --- |
| Internode length (cm) | | | | | | | |
| Rank 2 |  | 3.8 ± 0.3 cd | 4.1 ± 0.1 abc | 4.3 ± 0.2 ab | 4.0 ± 0.5 bcd | 4.5 ± 0.3 a | 3.6 ± 0.4 d |
| Rank 4 |  | 4.7 ± 0.5 cd | 5.2 ± 0.1 abc | 5.4 ± 0.3 a | 4.7 ± 0.3 bcd | 5.4 ± 0.6 ab | 4.1 ± 0.3 d |
| Rank 8 |  | 5.9 ± 0.3 b | 6.2 ± 0.1 b | 6.1 ± 0.3 b | 6.1 ± 0.3 b | 6.9 ± 0.9 a | 6.5 ± 0.3 ab |
| Rank 10 |  | 5.7 ± 1.1 a | 6.1 ± 0.3 a | 3.9 ± 1.0 bc | 5.6 ± 1.1 a | 3.3 ± 1.2 c | 5.5 ± 1.0 ab |
| Leaf area (cm^2^) | | | | | | | |
| Rank 2 |  | 39 ± 6 ab | 37 ± 1 b | 45 ± 2 a | 42 ± 7 ab | 42 ± 4 ab | 36 ± 7 b |
| Rank 4 |  | 73 ± 7 a | 72 ± 1 ab | 79 ± 4 a | 73 ±5 a | 74 ± 6 a | 62 ± 7 b |
| Rank 8 |  | 93 ± 3 a | 88 ± 3 abc | 92 ± 3 ab | 79 ± 7 cd | 82 ± 12 bcd | 74 ± 7 d |
| Rank 10 |  | 79 ± 7 a | 75 ± 3 ab | 57 ± 5 c | 70 ± 7 ab | 63 ± 2 bc | 65 ± 6 bc |
| Leaf inclination angle (°) | | | | | | | |
| Rank 2 |  | 28 ± 5 b | 33 ± 3 ab | 36 ± 2 a | 29 ± 6 ab | 34 ± 5 ab | 28 ± 6 b |
| Rank 4 |  | 28 ± 2 c | 32 ± 3 bc | 37 ± 2 ab | 31 ± 4 bc | 41 ± 3 a | 35 ± 4 bc |
| Rank 8 |  | 30 ± 9 abc | 29 ± 3 bc | 38 ± 3 a | 25 ± 4 c | 35 ± 5 ab | 24 ± 3 c |
| Rank 10 |  | 29 ± 5 d | 33 ± 3 cd | 49 ± 2 a | 36 ± 6 bc | 41 ± 2 b | 40 ± 8 bc |

Methods S1. Estimating leaf photosynthetic parameters

The combined measurement of gas exchange and chlorophyll fluorescence allows us to take into account the decrease of photosystem photochemical efficiency with the increasing of irradiance when estimating quantum efficiency of the leaf, which is more likely to happen in crops grown in relatively low light environment such as in the greenhouse (Yin *et al.*, 2014).

According to Genty *et al.* (1989), the apparent operating efficiency of photosystem II photochemistry (**_2_) was calculated as:

$\text{}_{2}=1-{F_{s}}/{F_{m}^{'}}$ (Eq. S1)

where *F*_s_ and *F*_m_’ were obtained directly from the combined gas exchange and chlorophyll fluorescence measurements.

According to Yin *et al.* (2014), the quantum yield of CO_2_ assimilation on the basis of incident light corrected for the decline of **_2_ with the increasing of light intensity (**_CO2LL(inc)_, mol CO_2_ mol^-1^ photon) was calculated as:

$\text{}_{CO2LL(inc)}=s^{'}\text{}_{2LL}/4$ (Eq. S2)

where *s*’ is a lumped parameter and **_2LL_ (mol e^−^ mol^-1^ photon) is the maximum value of **_2_. s’ was estimated as the slope between the linear regression below (Yin *et al.*, 2014):

$A=s^{'}\left( \frac{I_{inc}\text{}_{2}}{4} \right)-R_{d}$ (Eq. S3)

where *A* (*μ*mol CO_2_ m^-2^ s^-1^) is the net leaf photosynthetic rate; *I*_inc_ (*μ*mol m^-2^ s^-1^) is the incident irradiance; *R*_d_ (*μ*mol CO_2_ m^-2^ s^-1^) is the dark respiration. According to Yin *et al* (2009), **_2LL_ was estimated by fitting the measurements of **_2_ at different irradiance levels to the equation below:

$\text{}_{2}=(\text{α}_{2LL}I_{abs}+J_{2max}-\sqrt{\left( \text{α}_{2LL}I_{abs}+J_{2max} \right)^{2}-4\text{β}J_{2max}\text{α}_{2LL}I_{abs}})/(2\text{β}\text{}_{2}I_{abs})$ (Eq. S4)

where *J*_2max_ (*μ*mol m^-2^ s^-1^) is the total rate of electron transport passing PSII under saturating irradiance; *β* is the curvature factor; *ρ*_2_ is the factor of excitation partitioning to PSII (=*α*_2LL_/**_2LL_); *α*_2LL_ (mol e^−^ mol^-1^ photon) is the PSII photochemical efficiency under strictly limiting light on the basis of light absorbed by both PSI and PSII and is given by **_2LL_(1−*f*_cyc_)/(* *_2LL_/**_1LL_+1−*f*_cyc_), in which **_1LL_ (mol e^−^ mol^-1^ photon) is the photochemical efficiency of PSI and a value of 1.0 can be used for C_3_ species, and *f*_cyc_ is the fraction of cyclic electron transport in the total electron flux passing PSI and a value of 0.05 can be used for C_3_ species (Yin *et al.*, 2014).

With the **_CO2LL(inc)_ and *R*_d_ estimated above, the maximum leaf photosynthetic rate at the saturating incident irradiance level (*A*_max_, *μ*mol CO_2_ m^-2^ s^-1^) and the curvature factor of the light response curve (*θ*) were estimated by fitting the gas exchange measurements to the non-hyperbola rectangular equation below:

$A=\frac{\text{}_{CO2LL(inc)}I_{inc}+A_{max}-\sqrt{{(\text{}_{CO2LL(inc)}I_{inc}+A_{max})}^{2}-4\text{}A_{max}\text{}_{CO2LL(inc)}I_{inc}}}{2\text{}}-R_{d}$ (Eq. S5)

*References*

**Genty B, Briantais J-M, Baker NR**. **1989**. The relationship between the quantum yield of photosynthetic electron transport and quenching of chlorophyll fluorescence. *Biochimica et Biophysica Acta* **990**: 87–92.

**Yin X, Belay DW, van der Putten PEL, Struik PC**. **2014**. Accounting for the decrease of photosystem photochemical efficiency with increasing irradiance to estimate quantum yield of leaf photosynthesis. *Photosynthesis Research* **122**: 323–335.

**Yin X, Struik PC, Romero P, Harbinson J, Evers JB, Van Der Putten PEL, Vos J**. **2009**. Using combined measurements of gas exchange and chlorophyll fluorescence to estimate parameters of a biochemical C3 photosynthesis model: A critical appraisal and a new integrated approach applied to leaves in a wheat (Triticum aestivum) canopy. *Plant, Cell and Environment* **32**: 448–464.
